# Library size confounds biology in spatial transcriptomics data

**DOI:** 10.1186/s13059-024-03241-7

**Published:** 2024-04-18

**Authors:** Dharmesh D. Bhuva, Chin Wee Tan, Agus Salim, Claire Marceaux, Marie A. Pickering, Jinjin Chen, Malvika Kharbanda, Xinyi Jin, Ning Liu, Kristen Feher, Givanna Putri, Wayne D. Tilley, Theresa E. Hickey, Marie-Liesse Asselin-Labat, Belinda Phipson, Melissa J. Davis

**Affiliations:** 1 South Australian immunoGENomics Cancer Institute (SAiGENCI), Faculty of Health and Medical Sciences, The University of Adelaide, Adelaide, SA 5005 Australia; 2 Division of Bioinformatics, Walter and Eliza Hall Institute of Medical Research, Melbourne, VIC 3052 Australia; 3 Department of Medical Biology, Faculty of Medicine, Dentistry and Health Sciences, University of Melbourne, Parkville, VIC 3010 Australia; 4 The University of Queensland Fraser Institute, The University of Queensland, Woolloongabba, QLD 4102 Australia; 5 Melbourne School of Population and Global Health, School of Mathematics and Statistics, The University of Melbourne, Melbourne, VIC 3010 Australia; 6 Personalised Oncology Division, Walter and Eliza Hall Institute of Medical Research, Melbourne, VIC 3052 Australia; 7 Dame Roma Mitchell Cancer Research Laboratories, Adelaide Medical School, The University of Adelaide, Adelaide, SA Australia; 8 Department of Clinical Pathology, Faculty of Medicine, Dentistry and Health Sciences, University of Melbourne, Parkville, VIC 3010 Australia

## Abstract

Spatial molecular data has transformed the study of disease microenvironments, though, larger datasets pose an analytics challenge prompting the direct adoption of single-cell RNA-sequencing tools including normalization methods. Here, we demonstrate that library size is associated with tissue structure and that normalizing these effects out using commonly applied scRNA-seq normalization methods will negatively affect spatial domain identification. Spatial data should not be specifically corrected for library size prior to analysis, and algorithms designed for scRNA-seq data should be adopted with caution.

**Supplementary Information:**

The online version contains supplementary material available at 10.1186/s13059-024-03241-7.

## Background

After being crowned method of the year 2020 [1], spatial molecular technologies have seen significant advances with platforms providing diverse coverage of transcripts (100s to genome-wide) and spatial resolution of measurements (sub-cellular to 100s of cells) [2–5] through sequencing or imaging technologies. Spatial resolution of molecular measurements has enabled the study of diseases in their resident tissue microenvironment, thus, providing a more comprehensive view of disease systems [6]. The bioinformatics challenge posed by the increased scale and resolution of data has prompted abstraction of sub-cellular measurements to the cellular level [3, 4] by binning detections into segmented cellular boundaries [7]. Cellular abstraction enables the >1700 tools developed for the analysis of single-cell RNA sequencing (scRNA-seq) data to be applied to spatial molecular data [8]. However, this approach disregards spatial information and remains underpowered [3, 4]. Dedicated methods that incorporate spatial information are being developed [5, 7], but many analysis workflows still impose scRNA-seq assumptions.

One such assumption is that differences in the total number of transcripts detected/sequenced per cell (library size/total detections) represents technical variation that should be normalized out prior to downstream analysis. Normalization for library sizes originated from bulk RNA sequencing where samples were sequenced at varying depths [9]. The simplest method to account for library size in RNA-seq data is to divide each count by the total sequencing depth for that sample and multiply by a scalar to obtain counts per million (CPM) [10]. As this does not mitigate the effect of total sequencing depth in scRNA-seq experiments, new methods such as sctransform [11] and scran [12] have been proposed to reduce the impact of library size differences. In sub-cellular spatial molecular technologies, the unit of measurement is either a transcript detection or a sub-cellular spot; therefore, normalization at the cellular level is not as naturally motivated compared to bulk or scRNA-seq. Although cellular binning is not performed in Visium data, like other spatial molecular technologies, the proximity of spots/cells to neighboring spots/cells implies spatial autocorrelation resulting from biological dependence when spots/cells originate from the same tissue region. Additionally, unlike single-cell technologies that dissociate cells prior to sequencing, most spatial technologies measure the transcriptome while cells are embedded in tissue, and this could lead to differences in reagent permeability driven by tissue architecture. This would result in sampling differences across the tissue and subsequently library size differences. These effects are not accounted for in scRNA-seq normalization methods that are routinely applied to spatial data [2, 13]. The downstream effects of such normalization on some downstream analysis tasks have been shown on 10× Visium data but has not been extensively studied across other technologies [14].

### Results and discussion

**Fig. 1:**
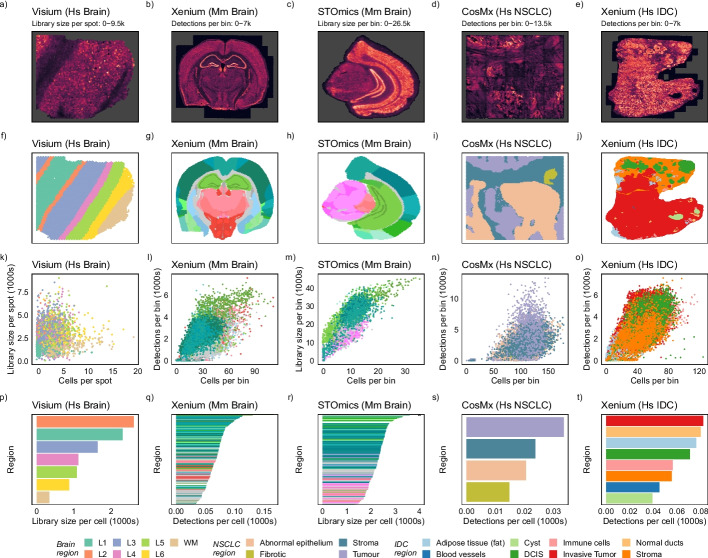
Detection density and total detections/library sizes are associated with biology consistently across different spatial molecular technologies, organs, and species. **a–d** Detection density per bin/spot plot for Visium dorsolateral prefrontal cortex (DLPFC), Xenium mouse brain, STOmics mouse brain, and CosMx non-small cell lung cancer (NSCLC) reveal tissue structure. **e–h** Regions annotated for each bin/spot using the Allen Brain Atlas for the mouse brain and manual annotation based on immunofluorescence markers of CosMx NSCLC. **i–l** Number of cells plot against the total detections/library sizes per bin/spot, colored by the tissue region, showing the region-specific relationship between cells and total detections/library sizes. **m–p** Average total detections/library sizes per cell for each region, computed as the sum of detections divided by the number of cells for each region, showing that related regions exhibit similar total detections/library sizes per cell. As the mouse brain datasets have over 100 regions annotated, color schemes from the Allen Brain Atlas are used where only larger structures are colored. (Note: truncated outlier marked by x)

Since library size can be a significant source of variability in single cell datasets, leading to clusters that capture library size differences rather than biological signals, we set out to investigate this effect in spatial data. Publicly available data from 25 tissue samples obtained using four different spatial technologies, including imaging- and sequencing-based methods, were used to study total detections/library sizes (Additional File 1: Supplementary Table 1) [3, 15–17]. We binned transcript detections into a hexagonal tessellation to explore total detections across space and visualized the density across bins/spots (Fig. 1a-e, Additional File 1: Supplementary Figure 1). We also annotated tissue regions in the datasets (see “Methods”) to assess library size associations with these regions (Fig. 1f-j, Additional File 1: Supplementary Figure 2). Visualizing the total detections/library sizes revealed tissue structure across brain and cancer datasets, including the layering of the cortex (darker greens in Fig. 1g-h), white matter (pinks in Fig. 1g-h), and hippocampus in the mouse brain datasets (brighter greens in Fig. 1g-h), and tumor regions in the non-small cell lung cancer (NSCLC) and breast intraductal carcinoma (IDC) dataset (purple and red regions in Fig. 1i-j respectively). Particularly, tumor regions had higher total detections per cell as expected since tumor cells are known to be transcriptionally more active than other cell types [14, 18, 19].

Our binning strategy allowed us to investigate total detections/library size without delving into cell boundary detection which is still an active area of research. However, this meant that each bin contained multiple cells; therefore, we had to relate the total detections/library sizes back to the number of cells. Visualizing total detections/library sizes against the number of cells revealed a linear dependency across all technologies with pronounced region-specific trends suggesting that cell density is not the only contributing factor to transcript detections (Fig. 1k-o, Additional File 1: Supplementary Figure 3). We estimated the average total detections/library sizes per cell for each region by dividing the total detections/library size of the region by the number of nuclei detected and found a clear region-specific effect in each dataset (Fig. 1p-t, Additional File 1: Supplementary Figure 4). Similar brain sub-structures, such as the different neuronal layers of the cortex (dark green bars), had similar average total detections/library sizes (Fig. 1q-r). Likewise, tumor regions had higher total detections per cell (Fig. 1s-t).

To assess the relationship between regions, the number of cells, and total detections/library sizes, we fitted a Poisson model to the binned data, treating all transcript detections as a spatial Poisson point process. The model included cell density, tissue region, and other technology-specific variables as covariates, and the interactions between all covariates were included in the model. The number of cells or the number of spots overlapping cells (STOmics) per bin explained the largest variance in library sizes, followed by the tissue region, across all technologies (Additional File 2). Even after accounting for the number of cells in each bin, there was a significant relationship between spatially defined regions and total detections/library sizes (tissue region *p*-values < 2 × 10^-308^, Additional File 2), which appears to be technology, species, and organ agnostic, and present across both healthy and disease systems.

**Fig. 2:**
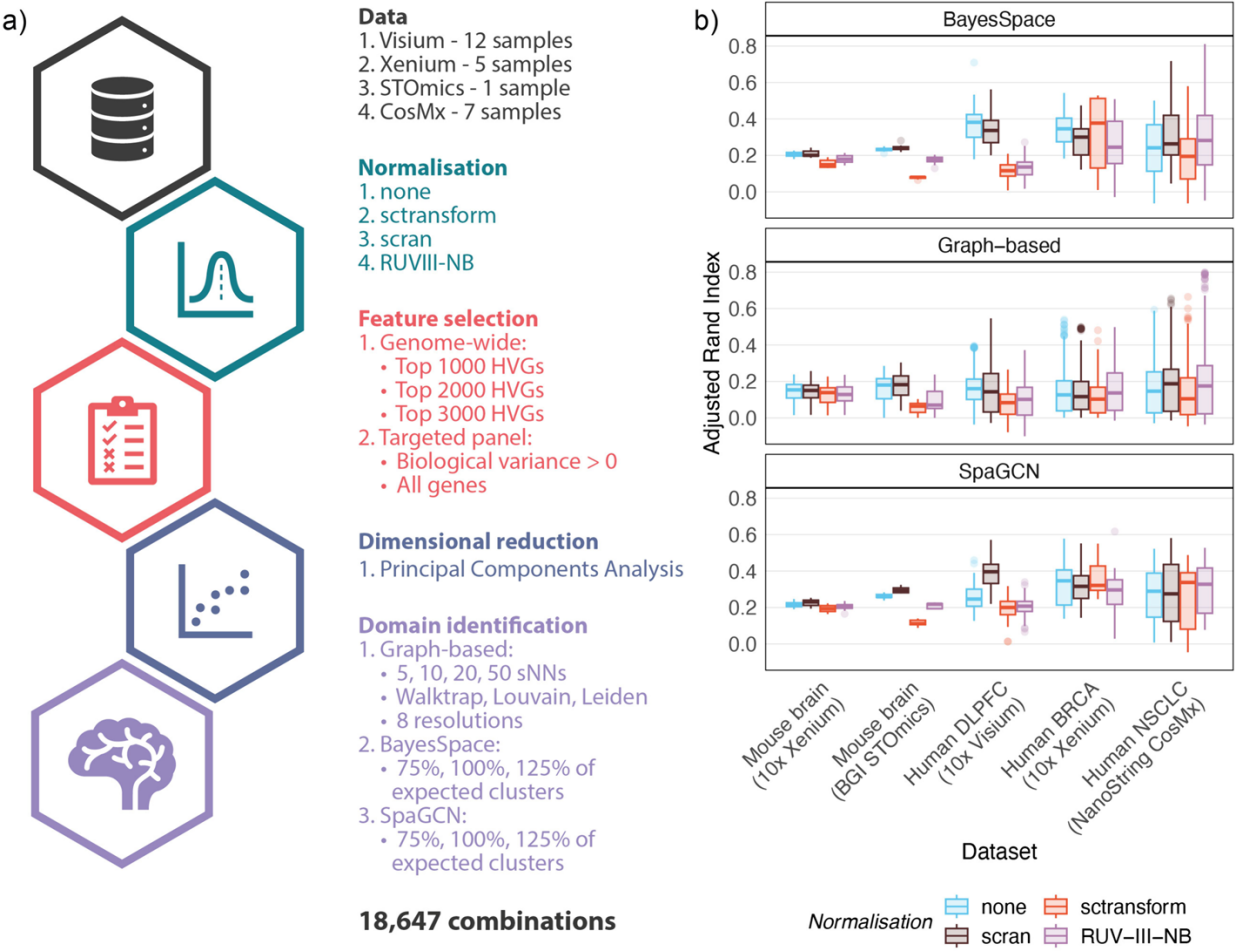
Normalization of total detections/library sizes results in poorer spatial domain identification using clustering approaches. **a** Schematic of the benchmark performed on 25 samples spanning four spatial transcriptomics technologies showing the parameter space explored when using a single-cell clustering pipeline, as well as two spatially aware methods to identify spatial domains. **b** The adjusted Rand index (ARI) obtained when different normalization strategies are applied on the different datasets using three different clustering methods: graph-based clustering, SpaGCN, and BayesSpace. Explicit library size normalization using sctransform results in poorer domain identification across most datasets, indicating that library size confounds biology in spatial transcriptomics datasets. Choice of normalization methods is dependent on the clustering algorithm and dataset type

The presence of region-specific total detections/library size effect implies that normalizing out total detections/library sizes could result in loss of information when attempting to identify spatial domains using clustering. Spatial domain identification in Visium data usually involves a standard single-cell clustering pipeline, where sctransform/scran normalization is applied [20]. Recent spatially aware domain identification methods such as BayesSpace [21] and SpaGCN [22], use the location information in conjunction with gene expression measurements. To evaluate the impact of normalization on these standard workflows without biases in parameter choice, a benchmark was set up to explore a large parameter space and test all combinations of parameters for each normalization strategy across 25 samples spanning all four technologies. Three normalization approaches (sctransform [11], scran [12], and RUVIII-NB [19]) were compared against not normalizing, but simply log-transforming the raw counts. In total, 18,647 different combinations were tested (Fig. 2a).

Performance on domain identification, as quantified by the adjusted Rand index (ARI), was strongly dependent on the choice of normalization methods (Fig. 2b). Library size normalization using sctransform resulted in poorer domain identification across most datasets and domain identification methods. Since sctransform’s effectiveness in removing library size effects results in poorer domain identification, our hypothesis of library size confounding biology in spatial transcriptomics datasets is confirmed. Though sctransform removes library size effects effectively, their confounding with biology results in removal of biological effects as well. Performance following RUVIII-NB, scran, and no normalization was primarily dependent on the clustering method: BayesSpace and graph-based clustering identified more accurate regions with unnormalized and scran normalized data while SpaGCN mostly favored scran normalization. We also observed dataset-specific trends where scran and no normalization resulted in better domain identification in normal tissues (human and mouse brains), while performance was less consistent in the cancer datasets, possibly due to the heterogeneity between samples (Additional File 1: Supplementary Figure 5). Next, we investigated the parameter combinations that produced the best domains for each of the 25 datasets (Additional File 1: Supplementary Table 2). Spatially aware clustering methods were the best at identifying domains for 21 of the 25 samples (with BayesSpace being the best for 11 of these and SpaGCN for 10). The normalization method that was better in most of these cases was scran (15/25 sample); however, normalization methods preferentially paired with clustering methods as seen in Fig. 2b. Finally, RUVIII-NB normalization produced the best domains for 5 of the 9 cancer datasets. Feature selection strategies varied across these best-case scenarios; however, dropping features with negative or zero biological variance estimates was beneficial for 11 of the 12 samples profiled using targeted panels (Xenium and CosMx).

Our analysis of spatial transcriptomic datasets from four different technologies and four different tissues shows that library size or total detections per cell significantly differ across tissue structures, representing real biology rather than technical variation. Technical effects such as differences in tissue permeability can explain variation in library sizes for technologies such as Visium and STOmics. However, as these differences are also driven by tissue architecture, they themselves confound biology and are difficult to decouple from truly technical variability. Similar observations have been made in scRNA-seq [19] and 10× Visium [14] data; however, this is the first time it has been rigorously tested across spatial molecular technologies. Normalizing this effect out will negatively impact spatial domain identification.

## Conclusion

We recommend carefully selecting when to normalize library sizes in spatial molecular data. For instance, library size normalization should not be performed prior to spatial domain identification but could be considered for other downstream analytical tasks that involve cross-sample comparisons/integration. Similar to a recent study that assessed the impact of normalization on differential expression analysis for marker detection [23], rigorous studies are needed to ascertain best practices for analyzing spatial transcriptomics datasets. Though not normalizing the data is better than sctransform normalization for clustering tasks, there is a need for new normalization methods that account for the unique properties of spatial data. We also emphasize caution when adopting ideas and tools from single-cell analysis into spatial molecular data as the assumptions of these methods may be violated for spatial data.

## Methods

### Hexagonal tessellation of sub-cellular localized data

We computed a hexagonal tessellation such that there were 100 hexagons along each axis. Since the areas profiled in the Xenium datasets were larger, the tessellation of these datasets contained 200 hexagons along each axis. This was preferred over a standard square grid as a hexagonal tessellation is less prone to edge effects [24]. Total detections/library size as well as the total number of cells were computed in each bin. The number of bins along each axis was heuristically selected such that each bin contained approximately 10 s of cells and 1000 s of detections to maintain comparability between datasets.

### Poisson model of binned counts

Points in space represent a Poisson point process therefore binning points will result in Poisson distributed count data. We model binned counts as a linear combination of the number of cells, the region types, and any technology specific technical covariates such as the number of DNA nanoball beads (BGI STOmics) and the field of view (NanoString CosMx). Generalized linear models with a log link function are used to perform the fit. All possible interactions between covariates were included in the models. A type II analysis of variance (ANOVA) [25] was performed on the covariates and their interactions within each model to assess their significance.

### Annotation of regions in spatial datasets

Mouse brain data from the Xenium and STOmics technologies were annotated by registering accompanying DAPI stained images to the “Nissl” channel of the common coordinates framework v3 (CCFv3) of the Allen Brain Atlas [26] using the Aligning Big Brains and Atlases (ABBA) plugin (v0.3.7) in Fiji (v1.53t) [27]. The BigWarp alignment pipeline was used to morph the DAPI image to the reference “Nissl” channel. The resultant hierarchical annotation was compressed such that the deepest layer of non-missing annotation was used to annotate each detection/DNA nanoball spot. This annotation resulted in the identification of 149–155 brain regions in the Xenium mouse brain dataset and 118 regions in the STOmics mouse brain dataset. Some regions have finer-resolution region annotations; therefore, each spot was annotated by the finest-resolution annotation available. Existing annotations for the non-small cell lung cancer (NSCLC) data were not used as these were derived using the transcriptomic measurements. Instead, we reannotated the data manually with QuPath (v0.3.2) [28] using the accompanying PanCK, CD3, CD45, and DAPI-stained images, thus producing annotations that were independent of the transcriptomics data. A total of six regions were annotated using these markers: Tumor, Stroma, Abnormal, Abnormal Epithelium, Necrotic, and Fibrotic. Xenium breast cancer data were annotated using the matched histopathology (H&E) image provided along with the dataset. The data were annotated for eight region types: ductal carcinoma in-situ (DCIS), invasive tumor, normal ducts, immune cells, cysts, blood vessels, adipose tissue, and stroma. Hexagonal bins were allocated to regions based on the predominant annotation of data points within the bin. Region annotations as well as estimates of cell numbers per spot for the Visium samples were available from the spatialLIBD R package [16].

### Pre-processing datasets for benchmarking

All samples were uniformly using the pipeline illustrated in Fig. 2a. Spots/bins lacking annotations, as well as those with total detections/library sizes less than 3 were removed. Following filtering, four different normalization strategies were applied: log transformation (no normalization), scran [12], sctransform [11], and RUVIII-NB [19]. Apart from RUVIII-NB which required negative control, all other methods were applied on target genes using default settings. For scran normalization, size factors estimated to be smaller than 10^-8^ were set to 10^-8^. RUV normalization was performed with the number of unwanted factors (K) set to 1. Single-cell housekeeping genes [29] are used as negative controls, except for datasets with less than 10 housekeeping genes available. For these, 10% of the genes were randomly selected as negative controls. Pseudo-replicates required by RUVIII-NB were defined by first selecting seed loci that were equidistantly spaced (approx. 0.5% of all loci). The 18 spots surrounding each seed locus (2nd degree neighborhood) were then considered to be distinct sub-populations and passed on as pseudo-replicates to RUVIII-NB. Pearson residuals produced by RUVIII-NB were used for downstream analysis.

Next, feature selection was performed by modeling gene variances using the scran R/Bioconductor package [30]. The top 1000, 2000, or 3000 highly variable genes were selected for datasets with genome-wide measurements (Visium and STOmics). For datasets obtained using targeted panels (Xenium and CosMx), either the full panel was selected or genes with variance estimates greater than 0 were chosen. Dimensional reduction was then performed using principal components analysis (PCA) to reduce dimensionality of the data to 50 principal components, thus retaining most of the information present in the data. Finally, data processed using all the above combinations were used to assess different clustering strategies.

### Domain identification benchmark

We evaluated a single-cell inspired graph-based clustering approach, as well as two spatially aware clustering methods: BayesSpace [21] and SpaGCN [22]. Shared nearest neighbor graphs were constructed for the graph-based approach with neighborhoods of size (k) 5, 10, 20, or 30. Next, community detection was performed using the walktrap, Louvain [31], or Leiden [32] approaches. Eight resolution parameters were explored for the latter two approaches (0.1, 0.225, 0.35, 0.475, 0.6, 0.725, 0.85, and 0.975). BayesSpace and SpaGCN were applied using the default settings recommended in their respective user guides. Both methods required the expected number of clusters to be specified. As domains were annotated in our study, we specified the number of unique spatial domain types. Though this information is available for our datasets, it is not always accurately known. Therefore, we also assessed performance of methods when the expected number of clusters is inaccurately over- or under-approximated by 25%. Clustering was performed using all combinations of parameters and methods, across all variants of pre-processed datasets. The Adjusted Rand index (ARI) was computed to evaluate clustering performance. The CellBench R/Bioconductor was used to execute the benchmark [33]. 

### Supplementary Information

**Additional file 1. **Additional figures to support the analyses in this study

**Additional file 2. **Results of Type II ANOVA tests on regression models of library size/total detections. (Df – degrees of freedom, Pr(>F) – *p*-value, Sum Sq – sum of squares).

**Additional file 3. **Review history

## Data Availability

All data used in this study, including region annotations (as GeoJSON files), are available on Zenodo (DOI: 10.5281/zenodo.7959786) [34] and are accessible through the SubcellularSpatialData R/Bioconductor data package [35]. SubcellularSpatialData provides access to the annotated transcript data as well as various functions to bin the data into cells, hexagonal bins, square bins, and regions. Reproducible code used to generate these analyses are available at https://davislaboratory.github.io/SpatialLibrarySizePaper [36]. The workflowr R package was used to ensure code reproducibility [37]. The code in this repository is covered by the MIT license and the written content is covered by a Creative Commons CC-BY license.
