# Supplementary material for: Library size confounds biology in spatial transcriptomics data
Source: Genome Biol. 2024 Apr 18;25:99. doi: 10.1186/s13059-024-03241-7 (PMC11025268; doi:10.1186/s13059-024-03241-7)
Supplement: Supplementary file 1 — Additional file 1. Additional figures to support the analyses in this study [file 13059_2024_3241_MOESM1_ESM.pdf]

Supplementary figures

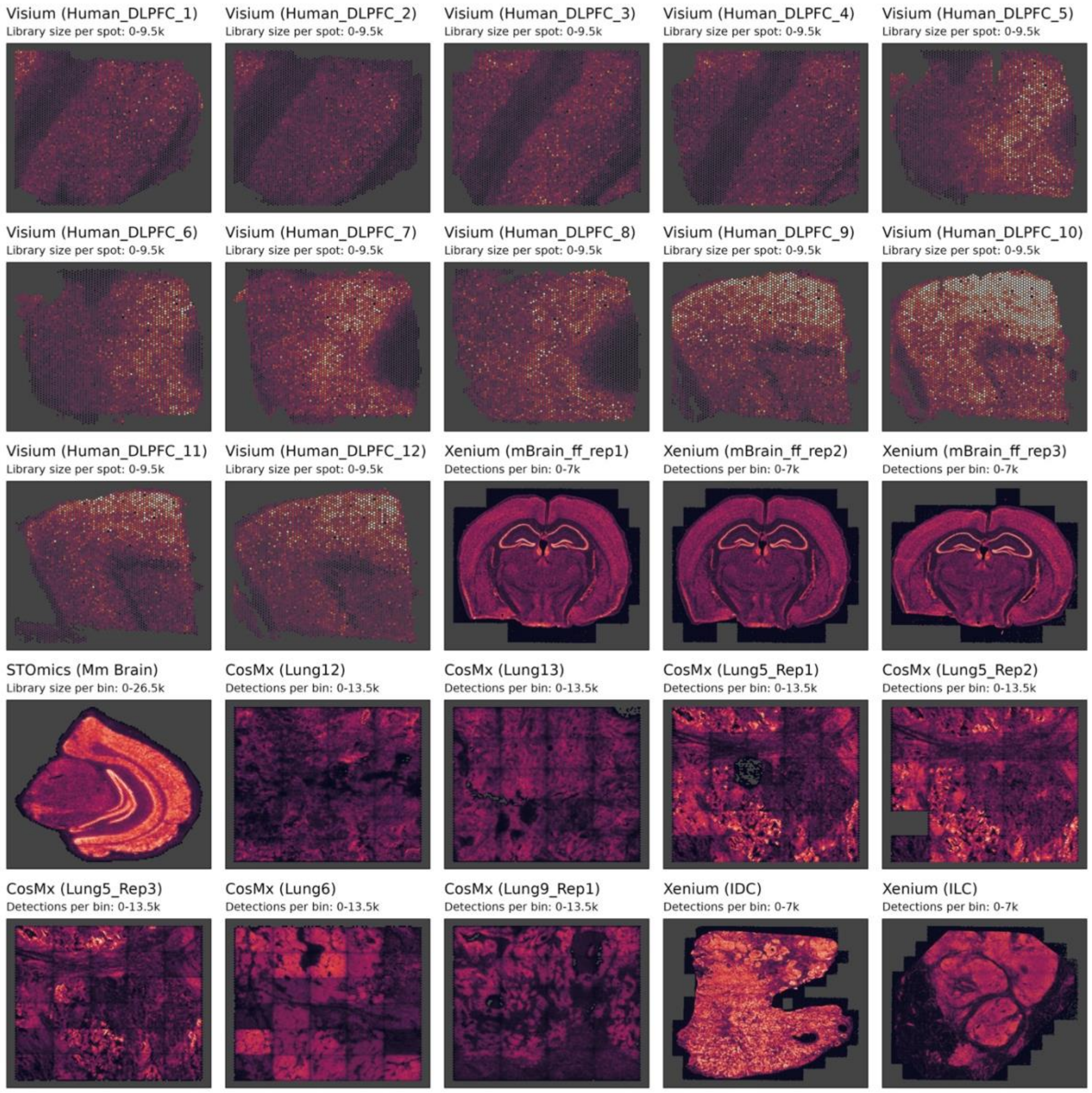

Supplementary Figure 1: *Detection density per bin/spot plot for Visium dorsolateral prefrontal cortex (DLPFC), Xenium mouse brain, STOmics mouse brain, CosMx non-small cell lung cancer (NSCLC), and Xenium human breast cancer, reveal tissue structure.*

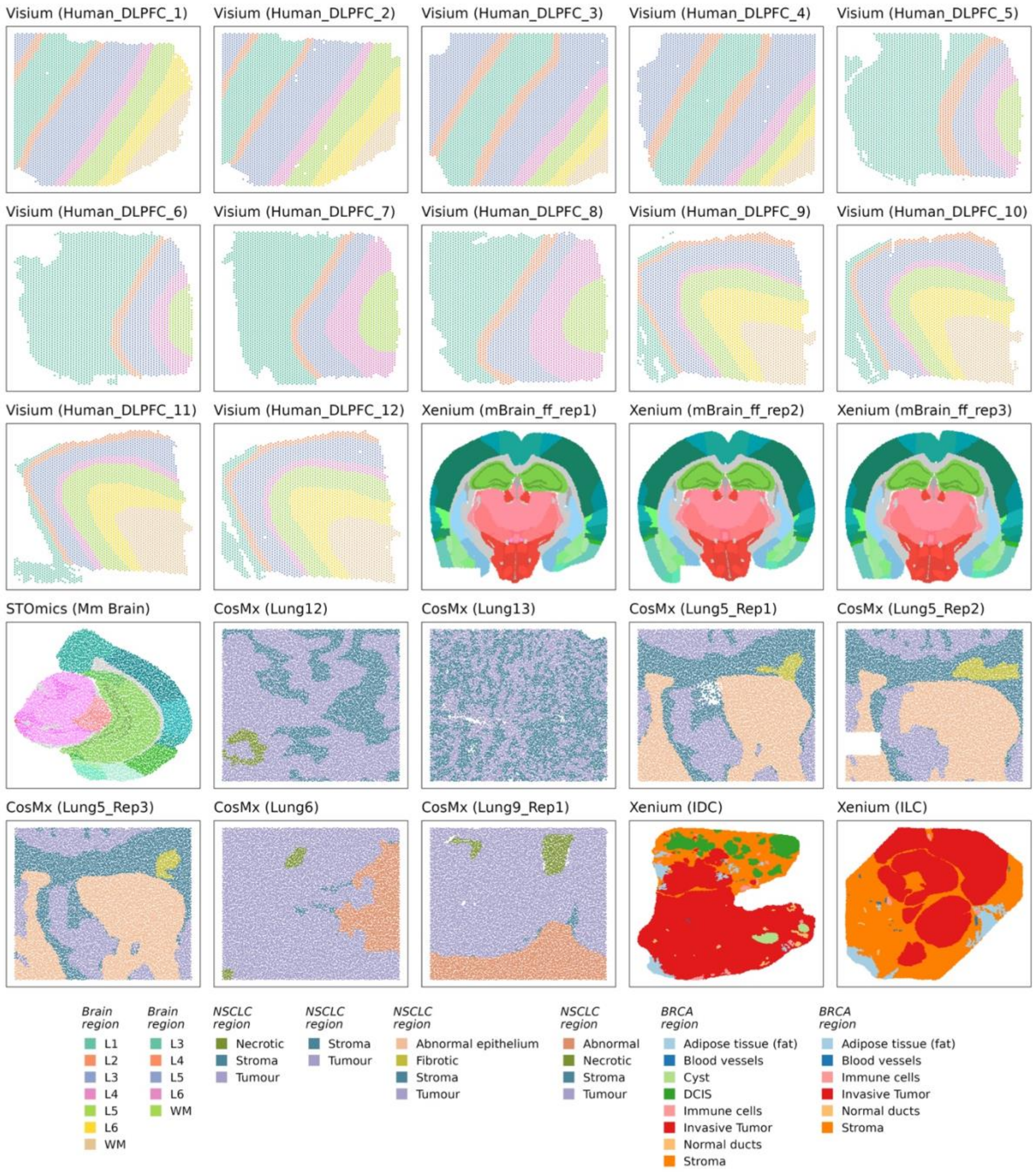

Supplementary Figure 2: *Regions annotated for each bin/spot using the Allen Brain Atlas for the mouse brain, manual annotation based on immunofluorescence markers of CosMx NSCLC, and manual annotation based on matched histology (H&E) images of Xenium breast cancer.*

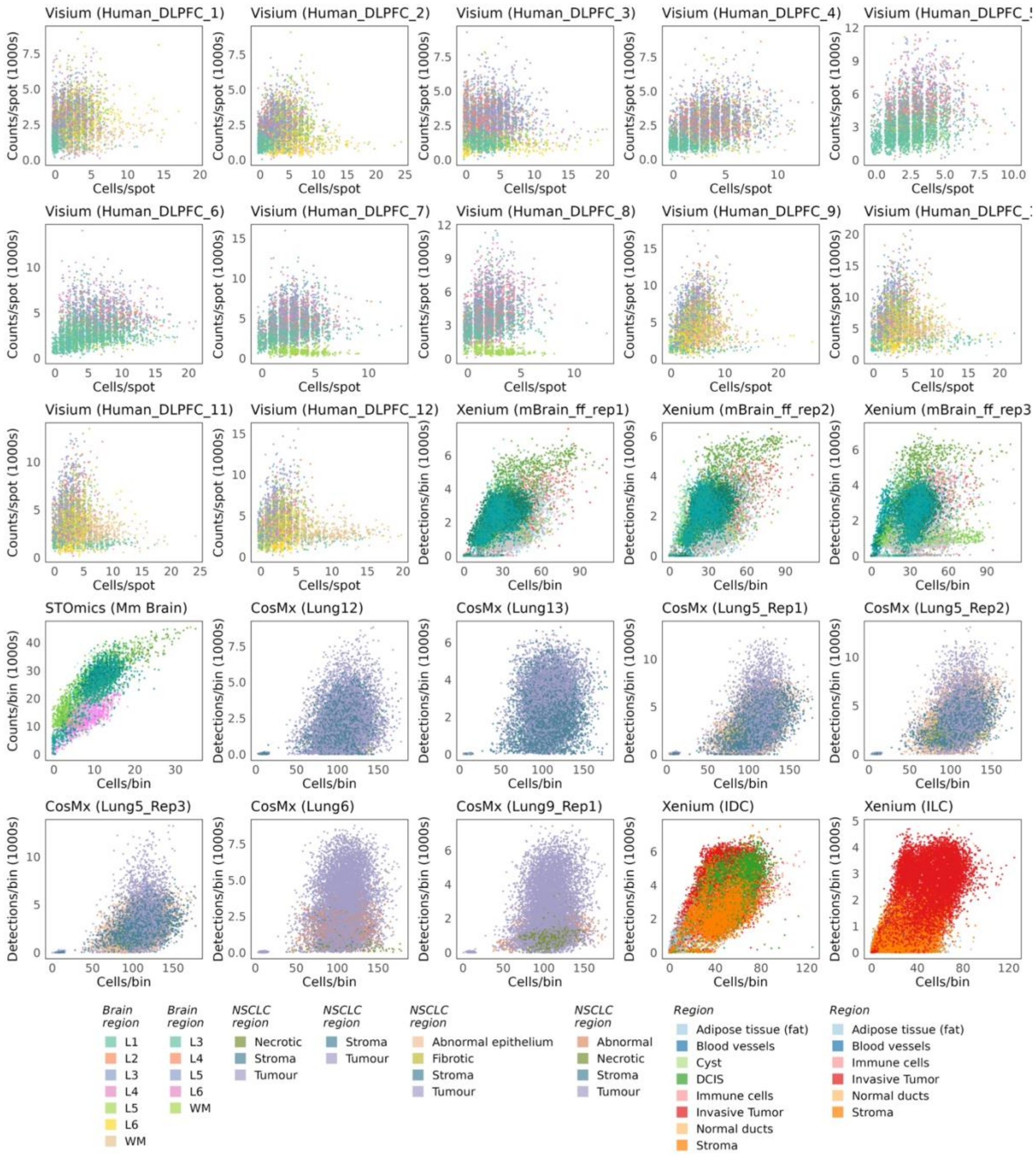

Supplementary Figure 3: *Number of cells plot against the total detections/library sizes per bin/spot, coloured by the tissue region, showing the region-specific relationship between cells and detections/counts.*

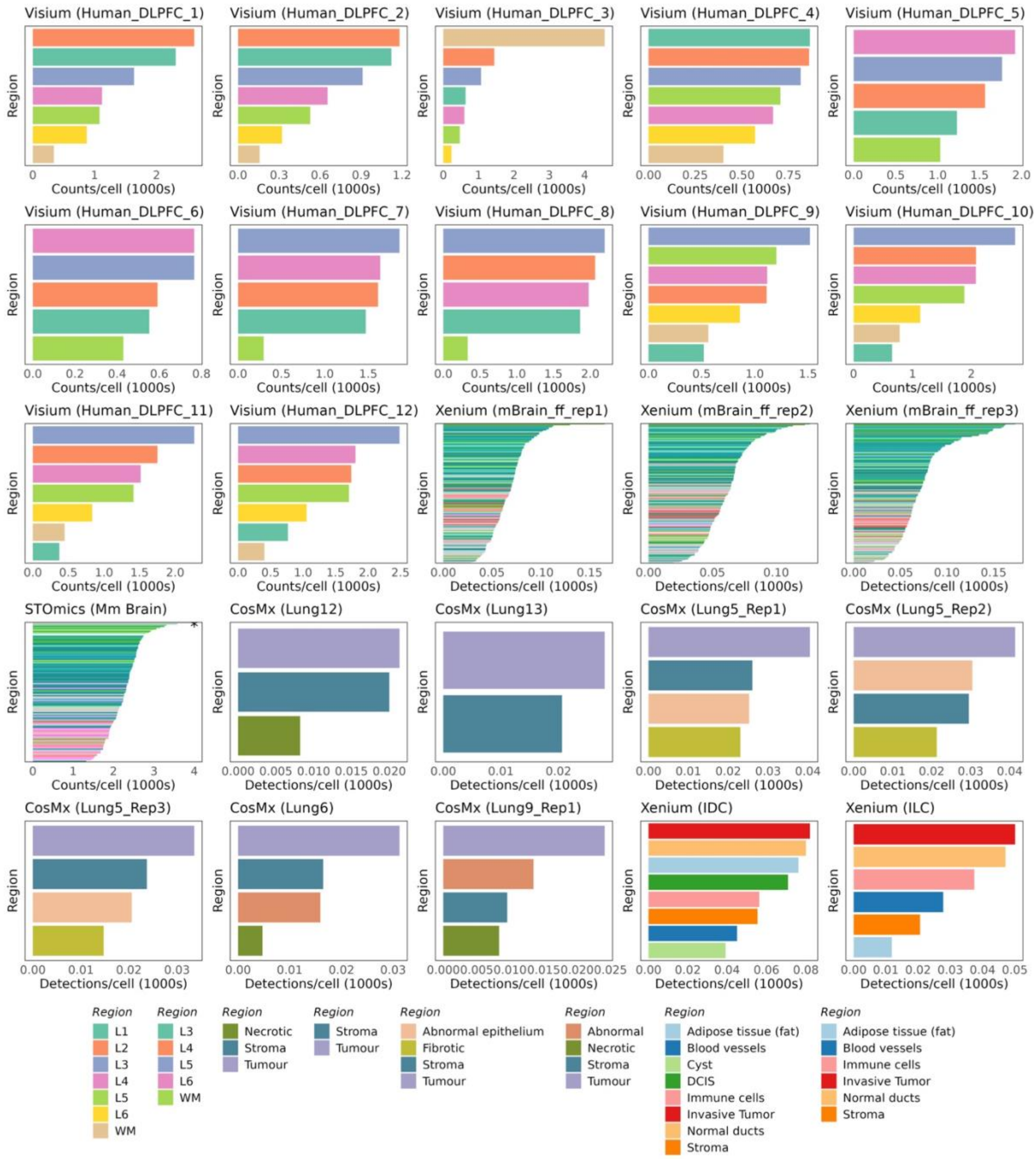

Supplementary Figure 4: *Average detections/library sizes per cell for each region, computed as the sum of detections divided by the number of cells for each region, showing that related regions exhibit similar total detections/library sizes per cell.*

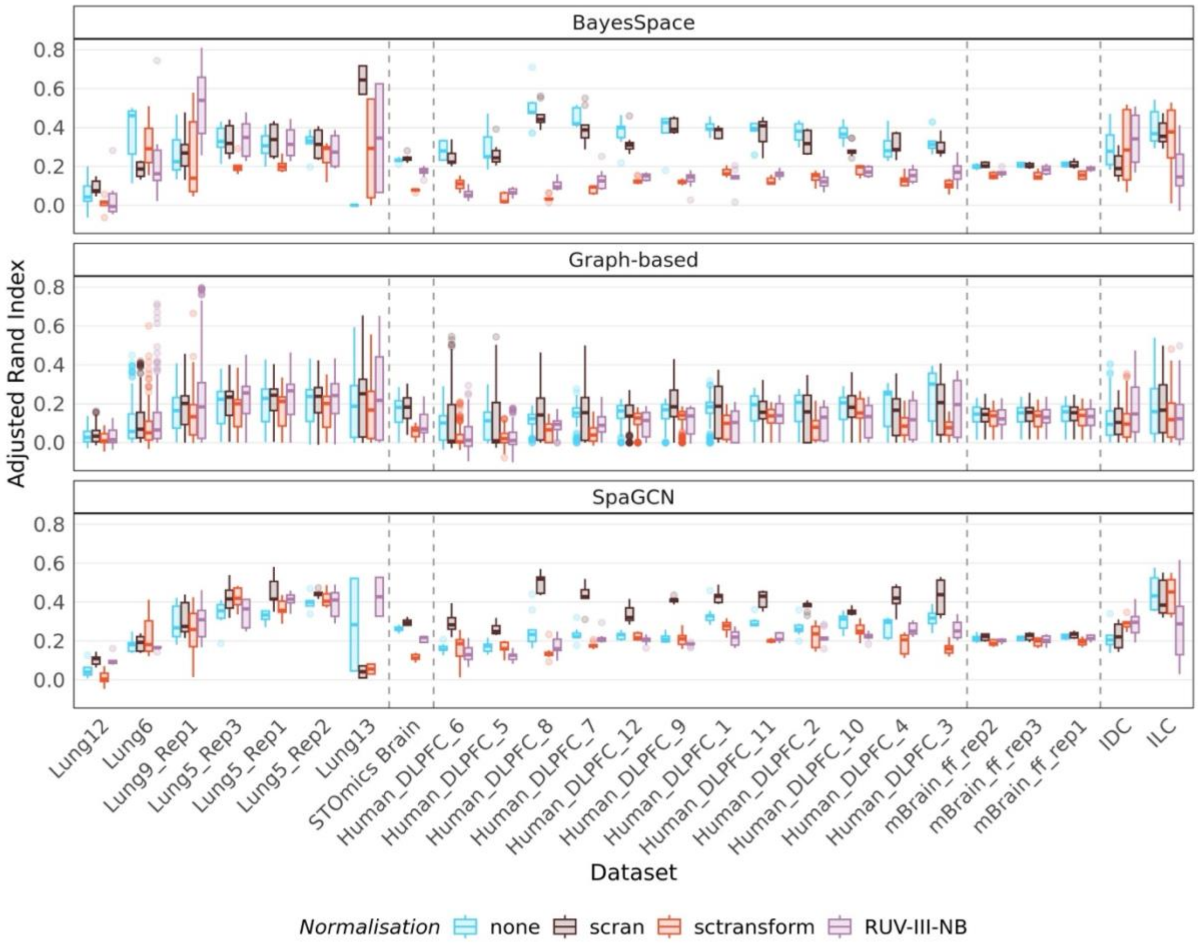

Supplementary Figure 5: *Impact of normalisation on domain identification of different datasets using 3 different clustering approaches: graph-based, SpaGCN, and BayesSpace. Dataset-specific as well as method-specific differences are observed (for instance, no normalisation and scran are better choices for visium data when using BayesSpace, but scran normalisation is preferred when using SpaGCN on the same data).*

Supplementary tables

Supplementary Table 1: *Spatial transcriptomics datasets used to study library size effects.*

Technology	Technology type	# samples	# genes	Total counts/ detections	Organism	Tissue	Source
10x Visium	Sequencing-based	12	33,538	9-22M	Homo sapiens	Dorsolateral prefrontal cortex	[1, 2]
10x Xenium	Imaging-based	3	248	58-62M	Mus musculus	Brain	[3]
10x Xenium	Imaging-based	2	380	53-101M	Homo sapiens	Breast cancer	[3]
NanoString CosMx	Imaging-based	7	960	25-40M	Homo sapiens	Non-small-cell lung cancer	[4]
BGI STOmics	Sequencing-based	1	26,177	134M	Mus musculus	Brain	[5]

Supplementary Table 2: *Parameter settings used to achieve the best domain identification for each dataset. sNN refers to the number of neighbours used to construct the shared nearest neighbour graph. NClusters refers to the number of expected clusters provided to the method.*

Sample	Normalisation Method	ARI	Clustering Method	Feature Method	Other Parameters
Human_DLPFC_8	none	0.709	BayesSpace	1000 HVGs	NClusters x 0.75
Human_DLPFC_7	scran	0.551	BayesSpace	3000 HVGs	NClusters x 0.75
Human_DLPFC_6	scran	0.546	Graph-based	3000 HVGs	Louvain (res = 0.35), sNN = 5
Human_DLPFC_5	scran	0.544	Graph-based	3000 HVGs	Louvain (res = 0.35), sNN = 5
Human_DLPFC_3	scran	0.529	SpaGCN	1000 HVGs	NClusters x 0.75
Human_DLPFC_4	scran	0.492	SpaGCN	1000 HVGs	NClusters x 0.75
Human_DLPFC_1	scran	0.490	SpaGCN	3000 HVGs	NClusters x 0.75
Human_DLPFC_12	none	0.464	BayesSpace	3000 HVGs	NClusters x 0.75
Human_DLPFC_11	scran	0.460	SpaGCN	2000 HVGs	NClusters
Human_DLPFC_9	scran	0.458	BayesSpace	3000 HVGs	NClusters
Human_DLPFC_10	none	0.443	BayesSpace	3000 HVGs	NClusters
Human_DLPFC_2	none	0.436	BayesSpace	3000 HVGs	NClusters x 0.75
mBrain_ff_rep3	scran	0.257	Graph-based	Variance > 0	Leiden (res = 0.475), sNN = 20
mBrain_ff_rep1	scran	0.253	SpaGCN	Variance > 0	NClusters x 1.25
mBrain_ff_rep2	scran	0.252	Graph-based	Variance > 0	Leiden (res = 0.475), sNN = 20
ILC	RUV-III-NB	0.617	SpaGCN	Variance > 0	NClusters x 0.75
IDC	sctransform	0.518	BayesSpace	Variance > 0	NClusters x 0.75
STOmics Brain	scran	0.324	SpaGCN	3000 HVGs	NClusters x 0.75
Lung9_Rep1	RUV-III-NB	0.811	BayesSpace	Variance > 0	NClusters x 0.75
Lung6	RUV-III-NB	0.744	BayesSpace	Variance > 0	NClusters
Lung13	scran	0.718	BayesSpace	All genes	NClusters x 1.25
Lung5_Rep1	scran	0.581	SpaGCN	Variance > 0	NClusters x 0.75
Lung5_Rep3	scran	0.539	SpaGCN	Variance > 0	NClusters
Lung5_Rep2	RUV-III-NB	0.490	SpaGCN	Variance > 0	NClusters
Lung12	RUV-III-NB	0.282	BayesSpace	Variance > 0	NClusters x 0.75

References

1. Maynard, K.R. et al. Transcriptome-scale spatial gene expression in the human dorsolateral prefrontal cortex. *Nat Neurosci* **24**, 425-436 (2021).
2. Pardo, B. et al. spatialLIBD: an R/Bioconductor package to visualize spatially-resolved transcriptomics data. *BMC Genomics* **23**, 434 (2022).
3. Genomics, X., Vol. 2023 (2023).
4. He, S. et al. High-plex imaging of RNA and proteins at subcellular resolution in fixed tissue by spatial molecular imaging. *Nat Biotechnol* (2022).
5. Chen, A. et al. Spatiotemporal transcriptomic atlas of mouse organogenesis using DNA nanoball-patterned arrays. *Cell* **185**, 1777-1792 e1721 (2022).
