# Supplementary material for: Library size confounds biology in spatial transcriptomics data
Source: Genome Biol. 2024 Apr 18;25:99. doi: 10.1186/s13059-024-03241-7 (PMC11025268; doi:10.1186/s13059-024-03241-7)
Supplement: Supplementary file 2 — Additional file 2. Results of Type II ANOVA tests on regression models of library size/total detections. (Df – degrees of freedom, Pr(>F) – p-value, Sum Sq – sum of squares). [file 13059_2024_3241_MOESM2_ESM.pdf]

| Sample | Sum Sq | Df | F value | Pr(>F) | Covariate | Technology |
|---|---|---|---|---|---|---|
| Human_DLPFC_1 | 34500.72 | 1 | 100.68 | 1.98E-23 | NCell | Visium |
| Human_DLPFC_1 | 117179397.43 | 7 | 48851.87 | 0.00E+00 | Region | Visium |
| Human_DLPFC_1 | 8845.69 | 6 | 4.30 | 2.48E-04 | NCell:Region | Visium |
| Human_DLPFC_2 | 2081.24 | 1 | 6.84 | 8.96E-03 | NCell | Visium |
| Human_DLPFC_2 | 92069861.97 | 7 | 43211.83 | 0.00E+00 | Region | Visium |
| Human_DLPFC_2 | 26463.78 | 6 | 14.49 | 1.93E-16 | NCell:Region | Visium |
| Human_DLPFC_3 | 7.34 | 1 | 0.02 | 8.80E-01 | NCell | Visium |
| Human_DLPFC_3 | 126789651.36 | 7 | 55808.31 | 0.00E+00 | Region | Visium |
| Human_DLPFC_3 | 40029.82 | 6 | 20.56 | 6.75E-24 | NCell:Region | Visium |
| Human_DLPFC_4 | 32441.75 | 1 | 99.77 | 2.96E-23 | NCell | Visium |
| Human_DLPFC_4 | 80591619.22 | 7 | 35406.66 | 0.00E+00 | Region | Visium |
| Human_DLPFC_4 | 5147.76 | 6 | 2.64 | 1.48E-02 | NCell:Region | Visium |
| Human_DLPFC_5 | 81563.47 | 1 | 153.18 | 1.72E-34 | NCell | Visium |
| Human_DLPFC_5 | 101041666.77 | 5 | 37952.89 | 0.00E+00 | Region | Visium |
| Human_DLPFC_5 | 16164.36 | 4 | 7.59 | 4.37E-06 | NCell:Region | Visium |
| Human_DLPFC_6 | 37491.09 | 1 | 68.34 | 1.93E-16 | NCell | Visium |
| Human_DLPFC_6 | 88332967.90 | 5 | 32204.90 | 0.00E+00 | Region | Visium |
| Human_DLPFC_6 | 84281.18 | 4 | 38.41 | 1.69E-31 | NCell:Region | Visium |
| Human_DLPFC_7 | 85864.99 | 1 | 165.99 | 2.94E-37 | NCell | Visium |
| Human_DLPFC_7 | 141814970.75 | 5 | 54828.36 | 0.00E+00 | Region | Visium |
| Human_DLPFC_7 | 27262.40 | 4 | 13.18 | 1.15E-10 | NCell:Region | Visium |
| Human_DLPFC_8 | 102953.38 | 1 | 206.08 | 1.42E-45 | NCell | Visium |
| Human_DLPFC_8 | 143497872.41 | 5 | 57447.41 | 0.00E+00 | Region | Visium |
| Human_DLPFC_8 | 23528.19 | 4 | 11.77 | 1.66E-09 | NCell:Region | Visium |
| Human_DLPFC_9 | 168425.63 | 1 | 219.50 | 2.99E-48 | NCell | Visium |
| Human_DLPFC_9 | 168867149.07 | 7 | 31439.23 | 0.00E+00 | Region | Visium |
| Human_DLPFC_9 | 40291.15 | 6 | 8.75 | 1.73E-09 | NCell:Region | Visium |
| Human_DLPFC_10 | 70677.51 | 1 | 81.42 | 2.89E-19 | NCell | Visium |
| Human_DLPFC_10 | 231980298.06 | 7 | 38179.13 | 0.00E+00 | Region | Visium |
| Human_DLPFC_10 | 87771.97 | 6 | 16.85 | 2.78E-19 | NCell:Region | Visium |
| Human_DLPFC_11 | 87182.45 | 1 | 134.54 | 1.47E-30 | NCell | Visium |
| Human_DLPFC_11 | 147515245.06 | 7 | 32520.23 | 0.00E+00 | Region | Visium |
| Human_DLPFC_11 | 80903.20 | 6 | 20.81 | 4.26E-24 | NCell:Region | Visium |
| Human_DLPFC_12 | 68630.43 | 1 | 108.44 | 5.07E-25 | NCell | Visium |
| Human_DLPFC_12 | 143893562.86 | 7 | 32481.28 | 0.00E+00 | Region | Visium |
| Human_DLPFC_12 | 126632.31 | 6 | 33.35 | 2.72E-39 | NCell:Region | Visium |
| mBrain_ff_rep1 | 3444356.83 | 1 | 15426.05 | 0.00E+00 | NCell | Xenium |
| mBrain_ff_rep1 | 326521854.93 | 140 | 10445.54 | 0.00E+00 | Region | Xenium |
| mBrain_ff_rep1 | 1115507.01 | 138 | 36.20 | 0.00E+00 | NCell:Region | Xenium |
| mBrain_ff_rep2 | 3614557.83 | 1 | 16128.12 | 0.00E+00 | NCell | Xenium |
| mBrain_ff_rep2 | 298501277.88 | 144 | 9249.38 | 0.00E+00 | Region | Xenium |
| mBrain_ff_rep2 | 1194535.82 | 142 | 37.54 | 0.00E+00 | NCell:Region | Xenium |
| mBrain_ff_rep3 | 1745971.08 | 1 | 6086.50 | 0.00E+00 | NCell | Xenium |
| mBrain_ff_rep3 | 317970313.77 | 144 | 7697.59 | 0.00E+00 | Region | Xenium |
| mBrain_ff_rep3 | 1051776.12 | 143 | 25.64 | 0.00E+00 | NCell:Region | Xenium |
| STOmics Brain | 216848735.03 | 118 | 30115.63 | 0.00E+00 | Region | STOmics |
| STOmics Brain | 13635312.04 | 1 | 223450.90 | 0.00E+00 | NSpots | STOmics |

| | | | | | | |
|---|---|---|---|---|---|---|
| STOmics Brain | 178506.05 | 110 | 26.59 | 0.00E+00 | Region:NSpots | STOmics |
| Lung12 | 687277.09 | 1 | 882.19 | 5.43E-187 | NCell | CosMx |
| Lung12 | 9758201.44 | 3 | 4175.23 | 0.00E+00 | Region | CosMx |
| Lung12 | 63117.98 | 1 | 81.02 | 2.58E-19 | FOV | CosMx |
| Lung12 | 9716.04 | 2 | 6.24 | 1.96E-03 | NCell:Region | CosMx |
| Lung12 | 69814.50 | 1 | 89.61 | 3.44E-21 | NCell:FOV | CosMx |
| Lung12 | 18098.54 | 2 | 11.62 | 9.13E-06 | Region:FOV | CosMx |
| Lung12 | 16647.14 | 2 | 10.68 | 2.31E-05 | NCell:Region:FOV | CosMx |
| Lung13 | 264911.33 | 1 | 432.71 | 2.24E-94 | NCell | CosMx |
| Lung13 | 11441740.90 | 2 | 9344.65 | 0.00E+00 | Region | CosMx |
| Lung13 | 5159.17 | 1 | 8.43 | 3.70E-03 | FOV | CosMx |
| Lung13 | 5877.71 | 1 | 9.60 | 1.95E-03 | NCell:Region | CosMx |
| Lung13 | 4650.18 | 1 | 7.60 | 5.86E-03 | NCell:FOV | CosMx |
| Lung13 | 7313.07 | 1 | 11.95 | 5.50E-04 | Region:FOV | CosMx |
| Lung13 | 8613.87 | 1 | 14.07 | 1.77E-04 | NCell:Region:FOV | CosMx |
| Lung5_Rep1 | 3119801.60 | 1 | 3213.47 | 0.00E+00 | NCell | CosMx |
| Lung5_Rep1 | 14001933.76 | 4 | 3605.58 | 0.00E+00 | Region | CosMx |
| Lung5_Rep1 | 175100.57 | 1 | 180.36 | 8.15E-41 | FOV | CosMx |
| Lung5_Rep1 | 22817.10 | 3 | 7.83 | 3.21E-05 | NCell:Region | CosMx |
| Lung5_Rep1 | 1341.21 | 1 | 1.38 | 2.40E-01 | NCell:FOV | CosMx |
| Lung5_Rep1 | 49412.00 | 3 | 16.97 | 5.43E-11 | Region:FOV | CosMx |
| Lung5_Rep1 | 22767.49 | 3 | 7.82 | 3.29E-05 | NCell:Region:FOV | CosMx |
| Lung5_Rep2 | 2077114.54 | 1 | 2503.86 | 0.00E+00 | NCell | CosMx |
| Lung5_Rep2 | 15581345.40 | 4 | 4695.64 | 0.00E+00 | Region | CosMx |
| Lung5_Rep2 | 186983.53 | 1 | 225.40 | 1.82E-50 | FOV | CosMx |
| Lung5_Rep2 | 19512.41 | 3 | 7.84 | 3.18E-05 | NCell:Region | CosMx |
| Lung5_Rep2 | 835.68 | 1 | 1.01 | 3.16E-01 | NCell:FOV | CosMx |
| Lung5_Rep2 | 76796.45 | 3 | 30.86 | 7.36E-20 | Region:FOV | CosMx |
| Lung5_Rep2 | 40856.41 | 3 | 16.42 | 1.21E-10 | NCell:Region:FOV | CosMx |
| Lung5_Rep3 | 2056427.37 | 1 | 2494.96 | 0.00E+00 | NCell | CosMx |
| Lung5_Rep3 | 9808764.80 | 4 | 2975.12 | 0.00E+00 | Region | CosMx |
| Lung5_Rep3 | 105280.25 | 1 | 127.73 | 1.82E-29 | FOV | CosMx |
| Lung5_Rep3 | 11572.50 | 3 | 4.68 | 2.86E-03 | NCell:Region | CosMx |
| Lung5_Rep3 | 10.53 | 1 | 0.01 | 9.10E-01 | NCell:FOV | CosMx |
| Lung5_Rep3 | 3612.38 | 3 | 1.46 | 2.23E-01 | Region:FOV | CosMx |
| Lung5_Rep3 | 18273.46 | 3 | 7.39 | 6.07E-05 | NCell:Region:FOV | CosMx |
| Lung6 | 467167.78 | 1 | 493.19 | 4.55E-107 | NCell | CosMx |
| Lung6 | 16171836.10 | 4 | 4268.12 | 0.00E+00 | Region | CosMx |
| Lung6 | 65955.69 | 1 | 69.63 | 7.96E-17 | FOV | CosMx |
| Lung6 | 60454.74 | 3 | 21.27 | 9.74E-14 | NCell:Region | CosMx |
| Lung6 | 35055.08 | 1 | 37.01 | 1.21E-09 | NCell:FOV | CosMx |
| Lung6 | 69811.80 | 3 | 24.57 | 7.70E-16 | Region:FOV | CosMx |
| Lung6 | 9264.03 | 3 | 3.26 | 2.06E-02 | NCell:Region:FOV | CosMx |
| Lung9_Rep1 | 430233.14 | 1 | 555.44 | 5.06E-120 | NCell | CosMx |
| Lung9_Rep1 | 9918301.48 | 4 | 3201.17 | 0.00E+00 | Region | CosMx |
| Lung9_Rep1 | 316468.66 | 1 | 408.57 | 2.50E-89 | FOV | CosMx |
| Lung9_Rep1 | 15867.87 | 3 | 6.83 | 1.36E-04 | NCell:Region | CosMx |
| Lung9_Rep1 | 66677.40 | 1 | 86.08 | 2.03E-20 | NCell:FOV | CosMx |

| | | | | | | |
|---|---|---|---|---|---|---|
| Lung9_Rep1 | 36195.56 | 3 | 15.58 | 4.15E-10 | Region:FOV | CosMx |
| Lung9_Rep1 | 721.96 | 3 | 0.31 | 8.18E-01 | NCell:Region:FOV | CosMx |
| IDC | 4870848.67 | 1 | 13529.67 | 0.00E+00 | NCell | Xenium |
| IDC | 363445710.13 | 8 | 126192.08 | 0.00E+00 | Region | Xenium |
| IDC | 244662.37 | 7 | 97.08 | 6.75E-141 | NCell:Region | Xenium |
| ILC | 2134088.04 | 1 | 5631.67 | 0.00E+00 | NCell | Xenium |
| ILC | 147858286.51 | 6 | 65030.88 | 0.00E+00 | Region | Xenium |
| ILC | 1073683.05 | 5 | 566.67 | 0.00E+00 | NCell:Region | Xenium |
