# Supplementary material for: Library size confounds biology in spatial transcriptomics data
Source: Genome Biol. 2024 Apr 18;25:99. doi: 10.1186/s13059-024-03241-7 (PMC11025268; doi:10.1186/s13059-024-03241-7)
Supplement: Supplementary file 3 — Additional file 3. Review history [file 13059_2024_3241_MOESM3_ESM.docx]

Review History

**First round of review**

**Reviewer 1**

**Were you able to assess all statistics in the manuscript, including the appropriateness of statistical tests used?**

Yes, and I have assessed the statistics in my report.

**Comments to author:**

The manuscript "Library size confounds biology in spatial transcriptomics data" addresses the importance of re-considering analysis of spatial transcriptomics (ST), specifically during normalization which is one the earliest and most important stages in common pipelines. The great value the authors add to the field with this work cannot be stressed enough, given the proliferation of studies using ST, which does not go in par with development of the basic statistical and methodological framework required to properly analyze this data. Considering that ST platforms are diverse in molecular and cellular resolution, I found valuable that the authors compared across several platforms (i.e., Visium, Xenium, Stereo-seq/STOmics, and CosMx). Their approach of binning the image-based detections was a simple, yet proper and ingenious approach. I also appreciate the efforts made by the authors to make code available via a GitHub website with explanations. It makes comprehension of the analysis much easier (although it seems like the code leading to clustering results was not included there. I strongly encourage the authors to add the full pipeline to the repository).

Upon reading the manuscript, I found a few main concerns which I consider should be addressed:

1. I failed to see in the methods how the users determined the number of nuclei in Visium data. This is an important point to clarify, as it's one of the aspects that makes comparable the results across technologies and supports the idea that more cellularity results in more counts.
2. For the analyses without normalization, does this mean raw integer counts? I believe this should be clarified to the reader who eventually would follow the suggestions made by the authors, as it is a rather unorthodox way to proceed with gene expression analysis.
3. The authors made clear that most of the library size variation results from biological sources, however it is quite possible that permeabilization (in the case of Visium and Stereo-seq) can contribute to the variation. Since the authors do not have wet-lab experimental results to show otherwise, I believe this point should be stressed in the Discussion.
4. How does differential gene expression analysis will be affected if counts are not normalized? DE analysis is the most likely step following domain detection, and not transforming the data could drastically affect its results and even violate the assumptions in many tests.
5. Some of those data sets contain domain-level annotations provided by those who generated the data. However, it looks like the authors re-annotated the data. Reasons should be provided as to why re-annotation was made. I suspect that it was due to the transcript binning.

Other minor concerns are:

6. The authors should emphasize more the fact that ARIs were not very high. The differences between pipelines are evident, but since highest ARIs were around 0.6 and it was the metric used to substantiate the suggestion of no-transformation or scran, it is an important thing to emphasize.
7. The authors say: "Similar brain sub-structures had similar average total detections/library sizes (Figures 1n-o)". I was confused about this statement, and because I saw that for Xenium and Stereo-seq, many more bars were shown than tissue domains. Then, after looking at the GitHub website, I noted that the authors perhaps were referring to neuron types, which can be collapsed into the domains shown in Figure 1. This should be detailed in the paper.
8. This could be obvious, but I think it should be noted: The authors subset the genes when testing the different pipelines. Since the smallest probe set is Xenium followed by CosMx, does this mean all genes were used for these data set? Or was other subset regime done on these two platforms?
9. Although not required to make the results of this work valid, more tumor samples would have added more value to this paper. As the authors pointed out, the tumor regions tend to have more counts, and this poses special challenges to tumor biology studies using ST. Brain samples tend to be more uniform (or at least I had that impression while looking the results of this paper)
10. Early in the text, the authors say "sub-cellular to 10s of cells", however GeoMx ROIs can contain hundreds of cells.
11. The authors use in some places "detections/library size" and in others "detections/counts". Uniformizing this could avoid confusions.

**Reviewer 2**

**Were you able to assess all statistics in the manuscript, including the appropriateness of statistical tests used?**

Yes, and I have assessed the statistics in my report.

**Comments to author:**

In this manuscript, Bhuva et al. demonstrate that the number of detected transcripts per cell/spot contains biologically relevant information in spatial transcriptomic (ST) data. While normalization to remove such variation within a dataset is commonplace in single-cell, and by extension, ST analysis pipelines, the manuscript shows that such normalization may actually harm the quality of the downstream analysis. Presenting results on datasets from four different ST technologies, the authors demonstrate this effect and suggest more care be taken in applying count normalization in ST analysis pipelines.

The manuscript is well-written and presented, and the message bears importance to the community creating and investigating ST data. With some changes, primarily to improve the clarity and robustness of the quantitative results, this manuscript will prove a valuable addition to the literature. Specific comments follow below.

Major Points
1. In Fig. 2b, due to the overlaying of all 3 conditions and the way in which max and median are shown, statements such as "Method A has the highest max, but not the highest median" are very difficult to visually determine from the figure. The max/median should be replaced with a standard visualization such as box and whiskers, and different conditions should not be horizontally overlayed.
2. The manuscript considers the different between "well-tuned parameters" (max) and more generally (median). However, it is important then to demonstrate whether the choice of parameters that constitutes "well-tuned" is consistent between datasets. Fig. 2 should also report the ARI performance on each dataset from the specific single choice of parameters that 1) maximizes overall performance across all datasets, 2) maximizes overall performance over all samples within the same dataset (for datasets with multiple samples).
3. While a full investigation into integrating multiple samples is presumably outside the scope of this paper, it should be reasonable to perform the described analysis pipeline on a merged dataset (e.g. of all DLFPC samples) and compare the normalization options. Knowing whether normalization is better than nothing here would be highly informative for further research into ST dataset integration.
4. While the manuscript uses region segmentation as the vehicle for comparison, it appears to only consider non-spatial community detection algorithms. This leaves unclear whether the observed phenomenon still appears when using natively spatial segmentation algorithms. The comparison should be expanded to include at least two spatial segmentation algorithms established in the literature, including at least one machine-learning and at least one non-machine-learning method.
5. The manuscript does not appear to contain specifics on how the pipeline described in Fig. 2a was performed, which makes it difficult to fully understand the significance of the results. This information should be available, including at least some information in the Methods section, and depending on space limitations, full information in SI/etc.
Minor Points
6. Fig. 2 as it appears in the combined PDF is very low resolution, although it was better when downloaded separately. It would be helpful if the authors could ensure the full resolution version appears in the combined PDF in future versions. Probably, avoiding JPEG format would be helpful.
7. It is unclear the point of "dimensional reduction" in Fig. 2a since there is only one option.
8. "Of note is that in general the ARI is not particularly high (max ARI = 0.6), indicating a need for improved analysis methods for spatial data." This comment should be revised in light of comment #4 (You don't aren't applying the existing improved spatial methods) and the fact that it is not given that the regions are perfectly detectable (that unsupervised clustering can reach near-1.0 ARI). For example, my experience with the DLFPC dataset suggests the theoretical upper limit for unsupervised clustering may be around 0.7-0.75 ARI.
9. Fig. 1m-p: Statistics of these numbers across all samples should be shown for each dataset (i.e. add error bars).
10. Fig1o - There's a little x or something in the top right of the figure - is that supposed to be there?

**Authors Response**

**Point-by-point responses to the reviewers’ comments:**

**Reviewer #1**

The manuscript "Library size confounds biology in spatial transcriptomics data" addresses the importance of re-considering analysis of spatial transcriptomics (ST), specifically during normalization which is one the earliest and most important stages in common pipelines. The great value the authors add to the field with this work cannot be stressed enough, given the proliferation of studies using ST, which does not go in par with development of the basic statistical and methodological framework required to properly analyze this data. Considering that ST platforms are diverse in molecular and cellular resolution, I found valuable that the authors compared across several platforms (i.e., Visium, Xenium, Stereo-seq/STOmics, and CosMx). Their approach of binning the image-based detections was a simple, yet proper and ingenious approach. I also appreciate the efforts made by the authors to make code available via a GitHub website with explanations. It makes comprehension of the analysis much easier (although it seems like the code leading to clustering results was not included there. I strongly encourage the authors to add the full pipeline to the repository).

Upon reading the manuscript, I found a few main concerns which I consider should be addressed:

1. I failed to see in the methods how the users determined the number of nuclei in Visium data. This is an important point to clarify, as it's one of the aspects that makes comparable the results across technologies and supports the idea that more cellularity results in more counts.

*These were available from the source datasets. We have explicitly mentioned this in text under the results section.*

**Changes to text (additions underlined, deletions strikethrough):**

***Methods: Annotation of regions in spatial datasets***

*Region annotations as well as estimates of cell numbers per spot for the Visium*

*samples were available from the spatialLIBD R package 23.*

2. For the analyses without normalization, does this mean raw integer counts? I believe this should be clarified to the reader who eventually would follow the suggestions made by the authors, as it is a rather unorthodox way to proceed with gene expression analysis.

*We have added a sentence in the text to clarify that for the case of no normalisation, counts are still transformed using the log function.*

**Changes to text (additions underlined, deletions strikethrough):**

***Methods: Annotation of regions in spatial datasets***

*To evaluate the impact of normalisation on this workflow without biases in parameter choice, a benchmark was set up to explore a large parameter space and test all combinations of parameters for each normalisation strategy across 25 samples spanning all four technologies. Three normalisation approaches (sctransform11,*

*scran^12^, and RUVIII-NB^16^) were compared against not normalising, but simply log-transforming the data.*

3. The authors made clear that most of the library size variation results from biological sources, however it is quite possible that permeabilization (in the case of Visium and Stereo-seq) can contribute to the variation. Since the authors do not have wet-lab experimental results to show otherwise, I believe this point should be stressed in the Discussion.

*We have included this statement in the discussion. It is true that library size effects can be a result of differences in tissue permeability, however, permeability itself could be different due to differences in tissue architecture therefore these technical effects themselves would confound biology. As such these effects would be difficult to decouple. We have added this point to the discussion.*

**Changes to text (additions underlined, deletions strikethrough):**

*Our analysis of spatial transcriptomic datasets from four different technologies and four different tissues shows that library size or total detections per cell significantly differ across tissue structures, representing real biology rather than technical variation. Technical effects such as differences in tissue permeability can explain variation in library sizes for technologies such as Visium and STOmics. However, as these differences are also driven by tissue architecture, they themselves confound biology and are difficult to decouple from truly technical variability.*

4. How does differential gene expression analysis will be affected if counts are not normalized? DE analysis is the most likely step following domain detection, and not transforming the data could drastically affect its results and even violate the assumptions in many tests.

*This is indeed the next step of standard analyses and would need to be researched extensively as part of a separate study. Following our preprint, other researchers have embarked on this question and studied the impact of normalisation in the context of differential expression analysis. Though this work has not undergone peer-review, we have cited it to guide readers to the article and assist them in making decisions regarding normalisation when performing DE analysis for marker detection.*

**Changes to text (additions underlined, deletions strikethrough):**

*For instance, library size normalisation should not be performed prior to spatial domain identification but could be considered for other downstream analytical tasks that involve cross-sample comparisons/integration. Similar to a recent study that assessed the impact of normalisation on differential expression analysis for marker detection^18^, rigorous studies are needed to ascertain best practices for analysing spatial transcriptomics datasets.*

5. Some of those data sets contain domain-level annotations provided by those who generated the data. However, it looks like the authors re-annotated the data. Reasons should be provided as to why re-annotation was made. I suspect that it was due to the transcript binning.

*The only dataset that has domain annotation coupled with the source dataset is the Visium human DLPFC data. We reused those annotations as well as pre-computed cell estimates per spot. As these were annotated by pathologists, they were robust independent annotations. We failed to clarify this in text and have subsequently added text in the methods section. All other datasets only had cell-level annotations that were derived from the transcriptomics measurements. To ensure a robust benchmark, we sought to annotate the other datasets using any additional independent information available with the data (e.g., immunofluorescence and histopathology images). This way, the annotations are obtained independently of the data and can form an unbiased truth set to benchmark against.*

**Changes to text (additions underlined, deletions strikethrough):**

***Methods: Annotation of regions in spatial datasets***

*Region annotations as well as estimates of cell numbers per spot for the Visium*

*samples were available from the spatialLIBD R package 23.*

Other minor concerns are:

6. The authors should emphasize more the fact that ARIs were not very high. The differences between pipelines are evident, but since highest ARIs were around 0.6 and it was the metric used to substantiate the suggestion of no-transformation or scran, it is an important thing to emphasize.

*Upon adding two new spatially aware domain identification methods (see Reviewer 2: comment 4), the ARIs are higher (max ARI is 0.81). These are high even when using unnormalised datasets as seen in the new Supplementary Figure 5 (see response to comment 4 for reviewer 2).*

7. The authors say: "Similar brain sub-structures had similar average total detections/library sizes (Figures 1n-o)". I was confused about this statement, and because I saw that for Xenium and Stereo-seq, many more bars were shown than tissue domains. Then, after looking at the GitHub website, I noted that the authors perhaps were referring to neuron types, which can be collapsed into the domains shown in Figure 1. This should be detailed in the paper.

*The Allen Brain Atlas provides hierarchical annotations for domains. As there are over 100 domains annotated in the data we investigated, we used the standard colour scale provided by the Allen Brain Atlas that focuses on colouring the higher-level domains. Subdomains then share the same colours (e.g., dark green for all regions of the cortex). Though subdomains of the cortex are primarily characterised by the different neuronal types, they are still subdomains that are not necessarily composed of pure cell types. As such, you will observe multiple dark green bars, each representing a subdomain of the cortex. We have clarified this information by adding a statement in the caption for Figure 1. We have also added an example for the cortex in the text to clarify that each bar represents a substructure.*

**Changes to text (additions underlined, deletions strikethrough):**

*Similar brain sub-structures, such as the different neuronal layers of the cortex (dark green bars), had similar average total detections/library sizes (Figures 1q-r).*

***Methods: Annotation of regions in spatial datasets***

*This annotation resulted in the identification of 149-155 brain regions in the Xenium mouse brain dataset, and 118 regions in the STOmics mouse brain dataset. Some regions have finer-resolution region annotations therefore, each spot was annotated by the finest-resolution annotation available.*

***Caption for Figure 1:*** *...As the mouse brain datasets have over 100 regions annotated, colour schemes from the Allen Brain Atlas are used where only larger structures are coloured.*

8. This could be obvious, but I think it should be noted: The authors subset the genes when testing the different pipelines. Since the smallest probe set is Xenium followed by CosMx, does this mean all genes were used for these data set? Or was other subset regime done on these two platforms?

*For these targeted panels, a different strategy was applied. Genes that had variances > 0 following the gene variance modelling were retained by default (Reviewer 2: comment 5). Additionally, we also repeated the benchmark with all panel genes. As such, two feature selection approaches were assessed for targeted panel datasets (variance > 0, and all genes), whereas three approaches were assessed for datasets with genome-wide measurements (n=1000, 2000, or 3000). We have added additional methods to accurately convey this information. The full addition to methods is detailed in the response to comment 5 from reviewer 2.*

**Changes to text (additions underlined, deletions strikethrough):**

***Methods: Pre-processing datasets for benchmarking***

*... Next, feature selection was performed by modelling gene variances using the scran R/Bioconductor package^26^. The top 1000, 2000, or 3000 highly variable genes were selected for datasets with genome-wide measurements (Visium and STOmics). For datasets obtained using targeted panels (Xenium and CosMx), either the full panel was selected or genes with variance estimates greater than 0 were chosen.*

9. Although not required to make the results of this work valid, more tumor samples would have added more value to this paper. As the authors pointed out, the tumor regions tend to have more counts, and this poses special challenges to tumor biology studies using ST. Brain samples tend to be more uniform (or at least I had that impression while looking the results of this paper)

*We have added two additional breast cancer samples: 1 intraductal carcinoma (IDC) and 1 intralobular carcinoma (ILC). These samples were profiled using the Xenium technology. Both these samples have also been included in the benchmark to enhance reproducibility of findings. In these new datasets, we see a similar profile of library sizes for cancerous regions. As the changes to text accompanying these new data are numerous but minor, we refrain from enlisting them here. However, these changes are in line with the previous text, therefore the message of the paper*

*remains the same, only strengthened with respect to cancer datasets. We have updated both figures as well as the supplementary figures.*

0. Early in the text, the authors say "sub-cellular to 10s of cells", however GeoMx ROIs can contain hundreds of cells.

*We have altered this as suggested.*

1. The authors use in some places "detections/library size" and in others "detections/counts". Uniformizing this could avoid confusions.

*We have unified these as per the reviewer’s suggestion. We now only refer to “total detections” or “library sizes” throughout the text.*

**Reviewer #2**

In this manuscript, Bhuva et al. demonstrate that the number of detected transcripts per cell/spot contains biologically relevant information in spatial transcriptomic (ST) data. While normalization to remove such variation within a dataset is commonplace in single-cell, and by extension, ST analysis pipelines, the manuscript shows that such normalization may actually harm the quality of the downstream analysis. Presenting results on datasets from four different ST technologies, the authors demonstrate this effect and suggest more care be taken in applying count normalization in ST analysis pipelines.

The manuscript is well-written and presented, and the message bears importance to the community creating and investigating ST data. With some changes, primarily to improve the clarity and robustness of the quantitative results, this manuscript will prove a valuable addition to the literature. Specific comments follow below.

Major Points

1. In Fig. 2b, due to the overlaying of all 3 conditions and the way in which max and median are shown, statements such as "Method A has the highest max, but not the highest median" are very difficult to visually determine from the figure. The max/median should be replaced with a standard visualization such as box and whiskers, and different conditions should not be horizontally overlayed.

*We have redesigned this figure as suggested while collapsing the individual samples within each dataset. This is to provide a clearer comparison of normalisation methods. We have also added an accompanying supplementary figure that displays the performance for each sample within each dataset separately (see response to comment 4 below for the accompanying changes to the manuscript).*

2. The manuscript considers the different between "well-tuned parameters" (max) and more generally (median). However, it is important then to demonstrate whether the choice of parameters that constitutes "well-tuned" is consistent between datasets. Fig. 2 should also report the ARI performance on each dataset from the specific single choice of parameters that 1) maximizes overall performance across all datasets, 2) maximizes overall performance over all samples within the same dataset (for datasets with multiple samples).

*We have now assessed this and have reported the results in the main text and as part of supplementary table 2. Details of the changes made to the manuscript are outlined in the response to comment 4 below. Briefly, we see that sctransform normalisation is the best choice for only 1 of the 25 samples studied. We also see that spatially aware methods are better for domain identification (21 of 25 samples) and that RUVIII-NB, a new normalisation method included in the benchmark, is a better choice for 5 of the 9 cancer samples. We did not see a specific feature selection strategy outperform others, however, targeted panel assays benefited from some degree of feature selection (even if it is to remove genes with zero or negative scran computed biological variance estimates). The settings used to achieve the best performance for domain identification are also presented in Supplementary Table 2.*

3. While a full investigation into integrating multiple samples is presumably outside the scope of this paper, it should be reasonable to perform the described analysis pipeline on a merged dataset (e.g. of all DLFPC samples) and compare the normalization options. Knowing whether normalization is better than nothing here would be highly informative for further research into ST dataset integration.

*The raw data (counts) have batch effects where the batch is the sample as demonstrated by the figure above (UMAP of log-transformed counts from the top 3000 highly variable genes). As such, batch integration needs to be performed prior to clustering. As the reviewer has pointed out, these data have already been integrated and integrated versions are available with the original publication. However, the specific pipeline the original authors used would influence the downstream clustering and would not allow us to assess the impact of normalisation. To do so, we will need to re-integrate the data using different pre-processing steps and different integration methods. Though this would be an interesting study, it would depart from the message of this paper, which is focused on assessing the impact of normalisation on downstream domain identification. Like the study that aimed to assess the impact of normalisation on differential expression analysis for marker detection (Reviewer 1: comment 4), an independent body of work would be required to extensively assess the impact of normalisation on batch integration and subsequent domain identification.*

4. While the manuscript uses region segmentation as the vehicle for comparison, it appears to only consider non-spatial community detection algorithms. This leaves unclear whether the observed phenomenon still appears when using natively spatial segmentation algorithms. The comparison should be expanded to include at least two spatial segmentation algorithms established in the literature, including at least one machine-learning and at least one non-machine-learning method.

*Upon the reviewer’s suggestion, we have included two new methods in the benchmark, BayesSpace and SpaGCN. These methods are good representations of the field and are the most widely adopted to date (based on citations). BayesSpace uses Markov random fields for clustering while SpaGCN uses graph convolutional networks. As such, they represent non-machine learning and machine learning based methods. We ran these methods with the default settings, however, on data pre-processed using all the combinations described in Figure 2a. Additionally, since these methods required the number of clusters to be specified, we permuted this value by either over- or under-specifying the number of clusters by 25%. This*

*represents the scenario where the user is not accurately able to approximate the number of expected clusters. The results are presented in the new version of Figure 2b which takes the focus away from individual samples, and instead summarises results at the dataset-level. We also show performance on individual samples through supplementary figure 5. We see that different clustering methods benefit from different normalisation strategies and that in almost all cases, explicit library size normalisation using sctransform results in the worst outcomes. Overall, as expected, spatially aware methods outperform the standard single-cell style graph-based pipeline. We thank the reviewer for recommending this additional work as it has greatly strengthened our manuscript.*

**Changes to text (additions underlined, deletions strikethrough):**

*Performance on domain identification, as quantified by the Adjusted Rand Index (ARI), was strongly dependent on the choice of normalisation methods (Figure 2b). Library size normalisation using sctransform resulted in poorer domain identification across most datasets and domain identification methods. Since sctransform’s effectiveness in removing library size effects results in poorer domain identification, our hypothesis of library size confounding biology in spatial transcriptomics datasets is confirmed. Though sctransform removes library size effects effectively, their confounding with biology results in removal of biological effects as well. Performance following RUVIII-NB, scran, and no normalisation was primarily dependent on the clustering method: BayesSpace and graph-based clustering identified more accurate regions with unnormalised and scran normalised data while SpaGCN mostly favoured scran normalisation. We also observed dataset-specific trends where scran and no normalisation resulted in better domain identification in normal tissues (human and mouse brains), while performance was less consistent in the cancer datasets, possibly due to the heterogeneity between samples (Additional File 1: Supplementary Figure 5). Next, we investigated the parameter combinations that produced the best domains for each of the 25 datasets (Additional File 1: Supplementary Table 2). Spatially aware clustering methods were the best at identifying domains for 21 of the 25 samples (with BayesSpace being the best for 11 of these and SpaGCN for 10). The normalisation method that was better in most of these cases was scran (15/25 sample), however, normalisation methods preferentially paired with clustering methods as seen in Figure 2b. Finally, RUVIII-NB normalisation produced the best domains for 5 of the 9 cancer datasets. Feature selection strategies varied across these best-case scenarios, however, dropping features with negative or zero biological variance estimates was beneficial for 11 of the 12 samples profiled using targeted panels (Xenium and CosMx).*

*~~The results showed that the median Adjusted Rand Index (ARI) across most
samples was higher when no normalisation or scran normalisation was performed
than when library size effects were explicitly removed using sctransform (Figure 2b).
This indicates that on average, better clustering is likely to be encountered without
normalisation or when normalising with scran. However, if parameters are tuned well
in the workflow, scran normalisation can result in better clustering, primarily for the
Visium samples (Figure 2b). These results highlight that total detections/library sizes
themselves contain region-specific information. Improved clustering on scran
normalised data compared to sctransform normalised data could be an indicator of
some residual library size effect following scran normalisation since scran does not
explicitly normalise out library size effects. We note that this comparison was performed without using spatial localisation information and it would be interesting to
see the impact of normalisation on methods dedicated to spatial molecular
technologies. Of note is that in general the ARI is not particularly high (max ARI =
0.6), indicating a need for improved analysis methods for spatial data.~~*

***Supplementary Figure 5***

5. The manuscript does not appear to contain specifics on how the pipeline described in Fig. 2a was performed, which makes it difficult to fully understand the significance of the results. This information should be available, including at least

***Figure 2***

some information in the Methods section, and depending on space limitations, full information in SI/etc.

*Following the reviewer’s recommendations, we have now added two additional methods subsections detailing the different pre-processing steps performed as well as the various combinations of clustering methods applied to pre-processed data.*

**Changes to text (additions underlined, deletions strikethrough):**

***Methods***

***Pre-processing datasets for benchmarking***

*All samples were uniformly using the pipeline illustrated in Figure 2a. Spots/bins lacking annotations, as well as those with total detections/library sizes less than 3 were removed. Following filtering, four different normalisation strategies were applied: log transformation (no normalisation), scran^12^, sctransform^11^, and RUVIII-NB^16^. Apart from RUVIII-NB which required negative control, all other methods were applied on target genes using default settings. For scran normalisation, size factors estimated to be smaller than 10-8 were set to 10-8. RUV normalisation was performed with the number of unwanted factors (K) set to 1. Single-cell housekeeping genes^25^ are used as negative controls, except for datasets with less than 10 housekeeping genes available. For these, 10% of the genes were randomly selected as negative controls. Pseudo-replicates required by RUVIII-NB were defined by first selecting seed loci that were equidistantly spaced (approx. 0.5% of all loci). The 18 spots surrounding each seed locus (2nd degree neighbourhood) were then considered to be distinct sub-populations and passed on as pseudo-replicates to RUVIII-NB. Pearson residuals produced by RUVIII-NB were used for downstream analysis.*

*Next, feature selection was performed by modelling gene variances using the scran R/Bioconductor package^26^. The top 1000, 2000, or 3000 highly variable genes were selected for datasets with genome-wide measurements (Visium and STOmics). For datasets obtained using targeted panels (Xenium, CosMx), either the full panel was selected or genes with variance estimates greater than 0 were chosen. Dimensional reduction was then performed using principal components analysis (PCA) to reduce dimensionality of the data to 50 principal components, thus retaining most of the information present in the data. Finally, data processed using all the above combinations were used to assess different clustering strategies.*

***Domain identification benchmark***

*We evaluated a single-cell inspired graph-based clustering approach, as well as two spatially aware clustering methods: BayesSpace^27^, and SpaGCN^28^. Shared nearest neighbour graphs were constructed for the graph-based approach with neighbourhoods of size (k) 5, 10, 20, or 30. Next, community detection was performed using the walktrap, Louvain^29^, or Leiden^30^ approaches. Eight resolution parameters were explored for the latter two approaches (0.1, 0.225, 0.35, 0.475, 0.6, 0.725, 0.85, and 0.975). BayesSpace and SpaGCN were applied using the default settings recommended in their respective user guides. Both methods required the expected number of clusters to be specified. As domains were annotated in our study, we specified the number of unique spatial domain types. Though this information is available for our datasets, it is not always accurately known.*

*Therefore, we also assessed performance of methods when the expected number of clusters is inaccurately over- or under-approximated by 25%. Clustering was performed using all combinations of parameters and methods, across all variants of pre-processed datasets. The Adjusted Rand Index (ARI) was computed to evaluate clustering performance.*

Minor Points

6. Fig. 2 as it appears in the combined PDF is very low resolution, although it was better when downloaded separately. It would be helpful if the authors could ensure the full resolution version appears in the combined PDF in future versions. Probably, avoiding JPEG format would be helpful.

*The PDF uses a version rendered by the submission system for review purposes. We have submitted a vector graphic of the image in our original submission and that should not pose any resolution issues in the final render from the journal. The main figures are all in vector format.*

7. It is unclear the point of "dimensional reduction" in Fig. 2a since there is only one option.

*Though only one option is included in the benchmark, we have it included in the figure to demonstrate the full analysis pipeline. The steps in Fig 2a represent the flow of data through the analysis pipeline. For completeness of this idea, we included dimensional reductions despite there being only one option.*

8. "Of note is that in general the ARI is not particularly high (max ARI = 0.6), indicating a need for improved analysis methods for spatial data." This comment should be revised in light of comment #4 (You don't aren't applying the existing improved spatial methods) and the fact that it is not given that the regions are perfectly detectable (that unsupervised clustering can reach near-1.0 ARI). For example, my experience with the DLFPC dataset suggests the theoretical upper limit for unsupervised clustering may be around 0.7-0.75 ARI.

*As expected by the reviewer, upon adding the spatially aware methods, the ARI is no longer so low for most of the studied datasets. We have subsequently removed the comment and have replaced the paragraph in the text as detailed in the response to comment 4 above.*

9. Fig. 1m-p: Statistics of these numbers across all samples should be shown for each dataset (i.e. add error bars).

*There are no error bars here as we are directly visualising the average number of transcripts detected per cell. As we do not estimate total detections/library sizes per cell, we are unable to estimate variation and subsequently visualise the error margins. To avoid introducing errors resulting from inaccurate cell segmentation across different technologies, we opted to estimate the average library size per cell within each region directly by dividing the total detections in each region by the number of detected nuclei in said region (excerpt from text below).*

*As the sample numbers across most datasets are small (Xenium brain – 3, xenium breast – 2, STOmics – 1) and the samples in some datasets are very heterogeneous (NSCLC: Lung 6 is a different sub-type and Lung 5 has 3 serial replicates),estimation error rates visualised through error bars would be misleading. The visium dataset is the only one that will benefit from such a visualisation. As a middle ground for this, we reported all results in Fig1 across the other datasets through supplementary figures 1-4. The matching results for Fig 1m-p are presented in supplementary figure 4.*

10. Fig1o - There's a little x or something in the top right of the figure - is that supposed to be there?

*This was an outlier that we truncated. We were meant to add a caption for this but missed it. We thank the reviewer for helping us identify this oversight. We have subsequently added this note to the caption.*

**Changes to text (additions underlined, deletions strikethrough): *Caption for Figure 1:*** *... (Note: outlier truncated and marked by x).*

**Second round of review**

**Reviewer 1**

The authors have addressed all the concerns appropriately. The study is now much stronger with the addition of data sets and clustering methods. I thank the authors for their efforts with this timely and thorough study.

The only minor detail I still would like to see explained is the re-annotation the data sets. I agree as to why only the annotations from the Visium brain cortex data set were used. However, the authors indicate that the other data sets did not have domain-level annotations. The NSCLC does have domain-level annotations (see "niche" column in the Giotto object metadata), but as assumed by authors, those were likely generated from gene expression alone. Some text indicating why those annotations were not used (i.e., more robust annotations made by the authors using mIF and stained tissue images) could clear the doubts. Of course, this could be only a personal curiosity, and hence I leave to the Editor to decide whether this is an important change to be made.

**Reviewer 2**

In this review, Bhuva et al. have effectively addressed the previous concerns by adding several new spatial segmentation methods to the benchmarking, restructuring the confusing elements of some of the plots to make them easy to understand, and adding necessary information regarding the pipeline so that the methodology of the manuscript is clear. With these changes, I feel that this manuscript is suitable for publication in Genome Biology and will make a valuable contribution to the study of spatial transcriptomics.

**Authors Response**

**Point-by-point responses to the reviewers’ comments:**

*The authors have addressed all the concerns appropriately. The study is now much stronger with the addition of data sets and clustering methods. I thank the authors for their efforts with this timely and thorough study.

The only minor detail I still would like to see explained is the re-annotation the data sets. I agree as to why only the annotations from the Visium brain cortex data set were used. However, the authors indicate that the other data sets did not have domain-level annotations. The NSCLC does have domain-level annotations (see "niche" column in the Giotto object metadata), but as assumed by authors, those were likely generated from gene expression alone. Some text indicating why those annotations were not used (i.e., more robust annotations made by the authors using mIF and stained tissue images) could clear the doubts. Of course, this could be only a personal curiosity, and hence I leave to the Editor to decide whether this is an important change to be made.*

In response to reviewer #1's comment, we have further clarified why we did not use
existing annotations available with the NSCLC dataset. We have added the the
following text to the Methods section: "Existing annotations for the non-small cell lung
cancer (NSCLC) data were not used as these were derived using the transcriptomic
measurements. Instead, we reannotated the data manually with QuPath (v0.3.2) [28]
using the accompanying PanCK, CD3, CD45 and DAPI stained images, thus
producing annotations that were independent of the transcriptomics data.".
